# Melatonin and Quercetin Co‐Treatment Attenuates Hepatic Damage in Diabetic Rats by Mitigating Oxidative Stress and Inflammation

**DOI:** 10.1002/jbt.70855

**Published:** 2026-04-22

**Authors:** Érique Ricardo Alves, Jaiurte Gomes Martins da Silva, Ismaela Maria Ferreira de Melo, Laís Caroline da Silva Santos, Marcelle Mariana Sales de França, Clovis Lapa Neto, Vanessa Bischoff Medina, Francisco de Assis Leite Souza, Leucio Duarte Vieira, Álvaro Aguiar Coelho Teixeira, Valéria Wanderley Teixeira

**Affiliations:** ^1^ Federal Rural University of Pernambuco (UFRPE) Recife Pernambuco Brazil; ^2^ Federal University of Alagoas (UFAL) Arapiraca Alagoas Brazil; ^3^ Federal University of Santa Maria (UFSM) Santa Maria Rio Grande do Sul Brazil; ^4^ Federal University of Pernambuco (UFPE) Recife Pernambuco Brazil

**Keywords:** experimental rats, flavonoids, hyperglycemia, indoleamine, inflammation, oxidative stress

## Abstract

Liver complications in diabetes are very common, contributing to a high mortality rate, making it essential to develop strategies to minimize this damage. We analyzed the effect of treatment with quercetin and melatonin on the liver of diabetic rats. The following groups were formed: GC—non‐diabetic rats; GD—diabetic rats; GDI—diabetic rats treated with insulin; GDM—diabetic rats treated with melatonin; GDQ—diabetic rats treated with quercetin; and GDQM—diabetic rats treated with quercetin/melatonin. Insulin (5 U/day), melatonin (10 mg/kg), and quercetin (40 mg/kg) were administered for 30 days after confirmation of diabetes. Weight, histology, immunohistochemistry (IL‐6, TNF‐α, and IL‐10), biochemistry (glycemia, AST, and ALT), morphometry, and oxidative stress were evaluated. Only animals in the GDI (89.50 ± 4.92 mg/dL) and GDM (90.75 ± 3.88 mg/dL) groups showed similar blood glucose levels to the GC group (87.50 ± 7.14 mg/dL). Liver weight was higher in the GD group (12.44 ± 1.55 g). In the GD group, TBARS levels were elevated (3.10 ± 0.93 nmol/mg), and there was a reduction in GSH (12.90 ± 1.03 nmol/mg). In GD, there was an increase in the percentage of lobular parenchyma (95.00 ± 0.90) and a reduction in non‐lobular parenchyma (5.00 ± 0.45). In the diabetic group, hydropic degeneration of hepatocytes and multifocal areas of coagulative necrosis were observed. AST (88.95 ± 11.45 U/L) and ALT (68.38 ± 1.79 U/L) were elevated. IL‐6 and TNF‐α were elevated in GD, while IL‐10 was reduced. It is concluded that treatment with melatonin and quercetin, alone or in combination, may be an adjunctive alternative in protecting the liver against diabetic complications in rats.

## Graphical abstract

Quercetin and melatonin, alone or combined, effectively protect the liver in diabetic rats. The antioxidants reduce hyperglycemia, oxidative stress, and inflammation, preventing hepatic damage.

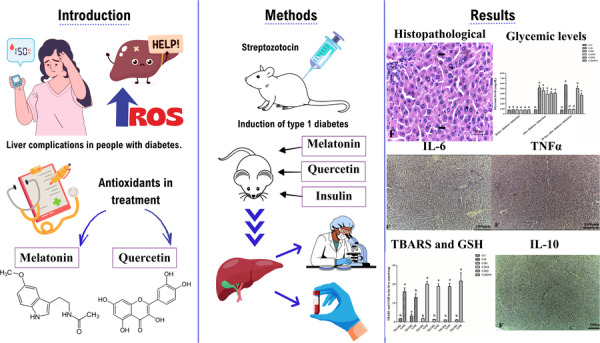

## 1 Introduction

Diabetes mellitus represents a major global health challenge due to its high prevalence, increasing incidence, and severe complications [1]. In 2024, the estimated global prevalence of type 1 diabetes mellitus was 9.2 million individuals, of whom 1.8 million were under 20 years of age [2]. Therefore, this disease requires substantial investment because of the essential needs related to prevention and disease management [3]. Consequently, numerous studies have investigated strategies aimed at mitigating the deleterious effects of diabetes [4].

Hyperinsulinemia, insulin resistance, lipotoxicity, oxidative stress, chronic inflammation, and advanced glycation end products are common consequences in diabetic patients and contribute to hepatic injury [5]. Accordingly, the liver is affected across several biochemical parameters, such as hepatic enzymes and cholesterol levels, as well as structural alterations, including inflammatory infiltrates, cellular hypertrophy, activated Kupffer cells, and bile duct dysplasia [6]. These changes may also include fatty degeneration, hepatitis, and hepatic fibrosis [7].

The standard treatment for type 1 diabetes is insulin therapy for glycemic control [8]. However, studies indicate that even after adequate glycemic control, metabolic damage caused by hyperglycemia continues to contribute to the development of complications [9]. In this context, one of the strategies proposed as an adjunctive treatment for diabetes is the control of oxidative stress [10]. Moreover, combinations of antioxidants have demonstrated potential to reduce oxidative stress, hyperglycemia, and the development of complications [11].

Melatonin is a hormone synthesized by the pineal gland, whose antioxidant activity has been widely reported in the literature [12]. Studies in humans and animal models have shown that this hormone may delay and/or reduce diabetic complications [13]. Another potent antioxidant is quercetin [14], a flavonoid naturally present in plants and not synthesized by the human body [15]. This compound exhibits antihyperglycemic, anti‐inflammatory, and anti‐apoptotic properties [16]. Its actions have been documented in the literature, indicating its potential as a therapeutic agent for diabetes by preventing and treating complications [17].

Melatonin and quercetin were selected based on their distinct yet potentially complementary mechanisms of action. Melatonin acts as a potent direct antioxidant, scavenging a wide range of reactive species, and indirectly by stimulating antioxidant enzymes such as glutathione peroxidase and superoxide dismutase [18]. Quercetin, a flavonol, in addition to its direct antioxidant capacity, is a known modulator of intracellular signaling pathways, such as the Nrf2 pathway, which regulates the expression of antioxidant genes, and the NF‐κB pathway, central to the inflammatory process [19, 20]. The hypothesis of a synergistic or additive effect with co‐administration is based on the possibility that melatonin acts primarily on the direct neutralization of free radicals and maintenance of mitochondrial function, while quercetin would strengthen long‐term endogenous antioxidant defenses via Nrf2 and modulate the inflammatory response. This combined approach could therefore offer broader and more effective protection against diabetes‐induced liver damage than either compound alone.

Although melatonin and quercetin individually show positive evidence in the treatment of diabetes, the effects of their simultaneous administration remain unclear. Therefore, the objective of this study was to investigate the effects of isolated and combined treatment with melatonin and quercetin on hepatic damage in streptozotocin‐induced diabetic rats.

## 2 Materials and Methods

### 2.1 Animals

Sixty male Wistar rats, 70 days old and weighing approximately 250 ± 30 g, from the Animal Facility of the Department of Animal Morphology and Physiology (DMFA) of the Federal Rural University of Pernambuco (UFRPE), were used. They were kept in an environment with controlled temperature (22 ± 1°C) and photoperiod (12 h light and 12 h dark) and under a regime of ad libitum feeding and water intake. The experimental protocol was submitted and approved by the Ethics Committee no.: 130/2019.

The sample size (*n* = 10 per group) was calculated based on an initial pilot study, considering a statistical power of 80% and a significance level of 5% (*α* = 0.05) to detect significant differences in blood glucose levels and oxidative stress markers between groups, using the standard formula for biological assays. The total number of 60 animals was considered the minimum necessary to guarantee the statistical validity of the results, minimizing the number of animals used, in line with the ethical principles of the 3Rs (Reduction, Replacement, and Refinement).

This study followed the ARRIVE (Animal Research: Reporting of In Vivo Experiments) guidelines for reporting research involving animals.

There was no animal mortality during the experimental period.

### 2.2 Experimental Groups

The sixty animals were divided into six experimental groups of 10 animals each. Group I (GC): rats without induced diabetes; Group II: rats with induced diabetes (GD); Group III: rats with induced diabetes treated with insulin (GDI); Group IV: rats with induced diabetes treated with melatonin (GDM); Group V: rats with induced diabetes treated with quercetin (GDQ); Group VI: rats with induced diabetes treated with melatonin and quercetin (GDQM). The substances were injected separately.

### 2.3 Vehicle Controls

To control for possible effects of the solvents used, additional groups of diabetic and non‐diabetic animals received only the vehicles (saline/ethanol solution for the intraperitoneal route and 2% dimethyl sulfoxide (DMSO) solution for the intragastric route) under the same administration regimes. Preliminary analysis showed no significant differences in the evaluated parameters between these groups and their respective controls (GC and GD), indicating that the solvents alone did not influence the results. Therefore, for the sake of clarity and animal number reduction, the data from these vehicle controls are not presented in the main figures and tables.

### 2.4 Diabetes Induction

Diabetes was induced by intraperitoneal administration of a solution of streptozotocin (Sigma Chemical Co., EUA, n° CAT. S0130) after a 14 h fast and confirmed on the fifth day after application. The streptozotocin was diluted in sodium citrate buffer at 10 mM and pH 4.5, in a single dose of 60 mg/kg of animal weight. Immediately after streptozotocin injection, the animals received a 5% glucose solution in their drinking water for the first 24 h to prevent severe hypoglycemia induced by massive pancreatic β‐cell destruction. Non‐diabetic animals (control group) received equivalent doses of saline solution in the same way, and 30 min after administration of streptozotocin, all animals were fed normally [21]. Only animals with blood glucose levels above 200 mg/dL (confirmed by Glucometer) (Accu‐Chek Active Kit, Roche Diagnostics, Germany), except the control group. All treatments began on the day the animals were confirmed to have diabetes.

### 2.5 Melatonin Treatment

Melatonin (Sigma Chemical Co., USA), N‐acetyl‐5‐methoxytryptamine (Sigma Chemical Co., St. Louis, USA. CAS N°:73‐31‐4) was administered in daily injections, for 30 days, intraperitoneally and always between 6:00 p.m. and 7:00 p.m., 10 mg/kg, which was dissolved in 0.2 mL of ethanol and diluted in 0.9 mL 0.9% NaCl. The 10 mg/kg dose was chosen as it is a commonly used experimental dosage well‐documented in the literature for its antioxidant and anti‐inflammatory potential in diabetes models [22, 23]. Administration in the dark mimics physiological secretion, which potentiates its metabolic effects [24, 25].

### 2.6 Quercetin Treatment

The flavonoid quercetin (Sigma Chemical Co., St. Louis, USA. CAS N°:117‐39‐5) was dissolved in a distilled water solution with a concentration of 2% DMSO [26]. This solution was subsequently homogenized in a vortex and then administered intragastrically for 30 days. The animals in the control group received only the DMSO solution. This dosage (40 mg/kg) was selected because it is within the safety limits of the substance and is widely reported in studies demonstrating its antioxidant and anti‐inflammatory effects [26, 27, 28, 29]. Furthermore, this dosage of 40 mg/kg simulates the daily intake of this flavonoid in humans [30, 31, 32].

### 2.7 Insulin Administration

Insulin (Eli Lilly and Co., USA. CAS N°:11061‐68‐0) was administered subcutaneously for 30 days at a dose of 5 U/day, being two units of insulin at 10:00 a.m., he has three units remaining at 7:00 p.m. [33]. Animals were monitored for signs of hypoglycemia after administration.

### 2.8 Blood Glucose Levels

The animals' blood glucose levels were monitored during the experimental period, measured using a Glucometer Kit (Accu‐Chek Active Kit, Roche Diagnostics, Germany), in the moments before induction, after confirmation of diabetes (5 days after induction) and 30 days of treatments with insulin, melatonin, and quercetin.

### 2.9 Liver Collection

Rats were anesthetized intramuscularly with ketamine (80 mg/kg) and xylazine (6.0 mg/kg) for liver collection. Following the abdominal incision and organ removal, the animals were euthanized with an intramuscular dose of the same anesthetic combination supplemented with intraperitoneal thiopental (100 mg/kg). Immediately after collection, each liver was washed in ice‐cold saline, gently dried with filter paper, and weighed on an analytical balance (Marte, Model AY220) to obtain the absolute organ weight.

Liver fragments were fixed in buffered formalin for 48 h. They were then dehydrated in increasing concentrations of ethyl alcohol, diaphanized in xylene, impregnated, and embedded in paraffin. The paraffin blocks were cut with a Minot microtome (Leica RM 2035, Leica Biosystems, Germany) set at 5 μm. The sections obtained were placed on slides previously smeared with Mayer's albumin and kept in an oven at 37°C for 24 h to dry and adhere. Subsequently, the sections were stained with hematoxylin and eosin (HE) and analyzed and photographed using an OLYMPUS BX‐49 and OLYMPUS BX‐50 light microscope, respectively.

### 2.10 Analysis of Lipid Peroxidation (TBARS) and Reduced Glutathione (GSH)

Oxidative stress was estimated by evaluating lipid peroxidation (TBARS) and GSH levels in the livers of the animals. The liver tissue was macerated in a solution of 150 mM KCl and 3 mM ethylenediaminetetraacetic acid (EDTA) (1 g of tissue: 5 mL). Lipid peroxidation was evaluated by the method of Ohkawa et al. [34] for measuring substances reactive to thiobarbituric acid in the homogenate, with the standard curve drawn using 1,1,3,3‐tetraethoxypropane (TEP) and the absorbance of the organic phase measured at 535 nm [35]. GSH levels were evaluated by quantifying non‐protein sulfhydryl groups [36] in the supernatant of the samples, which were subjected to protein precipitation by the addition of trichloroacetic acid. l‐cysteine was used to construct the GSH standard curve, and absorbance was measured at 412 nm. TBARS and GSH levels were corrected for protein content, measured by the Folin phenol method [37, 38, 39].

### 2.11 Morphometric Analysis

A morphometric study was performed according to the methodology described by Engelman et al. [40]. The proportion between the non‐lobular and lobular parenchyma of the liver of rats in the experimental groups was determined by stereological methods, using a grid with 100 test points. The count was made on three slides, so that 10 fields were counted using a ×40 objective, totaling 3000 points per group. Similarly, Kupffer cells were quantified in hematoxylin‐eosin preparations, in which the elongated and characteristic nucleus of the cells can be seen in relation to the large and rounded nuclei of the hepatocytes.

### 2.12 Biochemical Analysis

Samples were collected 10 and 30 days after treatments. For this, the rats were immobilized in containment, mechanically, and blood was collected by puncture of the lateral caudal vein using a catheter, and on the last day, through cardiac puncture (24G) [41]. After refrigerated centrifugation, the plasma was stored in a microtube Eppendorf, in duplicate, and frozen at −20°C until the time of dosages [42]. Liver function was determined by the activity of transaminases, alanine aminotransferase (ALT), and aspartate aminotransferase (AST). For this, reagent 1 was added to a glass tube, and subsequently this tube was placed in a water bath for 3 min at 37°C, then the sample was added and homogenized and returned to the water bath at 37°C for 30 min. Then reagent 2 was added and once again it was homogenized and left at room temperature for 20 min, the final step consisted of adding the working reagent and vortexing, followed by reading the absorbance in the spectrophotometer (UV‐1600PC, VWR International, USA) at 505 nm. To calculate the results, use the calibration curve that accompanies the kit. The procedures were the same for reading both AST and ALT, but with different kits.

### 2.13 Immunohistochemical Analysis (IL‐6, IL‐10, and TNF‐α)

Silanized slides were deparaffinized and rehydrated in xylene and alcohol, respectively. Antigen retrieval was performed using a citrate buffer solution (pH 6.0) at high temperature in the microwave for 5 min. Endogenous peroxidase was inhibited using a hydrogen peroxide solution (3%) in methanol. The nonspecific antigen‐antibody reaction was blocked by incubating the slides in PBS and 5% bovine serum albumin (BSA) for 1 h. All antibodies (Santa Cruz Biotechnology Inc., Santa Cruz, CA, USA) were diluted in PBS/1% BSA and incubated overnight in the refrigerator. Subsequently, the slides were treated with the secondary antibody, Histofine, for 30 min. The antigen‐antibody reaction was observed through a brown precipitate after application of 3,3‐diaminobenzidine for 4 min and counterstained with hematoxylin. The images were captured using a Sony, coupled to an Olympus Bx50 microscope, and submitted to the Gimp 2.0 application for quantification using an RGB Histogram (Red‐Green‐Blue). The pixel tones in the image range from 0 to 255, where tone 0 represents absolute darkness (lowest luminescence), while tone 255 represents absolute white (highest luminescence). Two slides from each group were used, where four fields/slide were photographed for marking measurements [43, 44].

### 2.14 Statistical Analysis

Statistical analysis was performed using GraphPad Prism 8.01 software (GraphPad Software, San Diego, CA, USA). Initially, data normality was assessed by the Shapiro−Wilk test. As all data showed a normal distribution, comparisons between multiple groups were performed using one‐way analysis of variance (ANOVA), followed by the Tukey−Kramer post hoc test for multiple comparisons. Data were expressed as mean ± standard deviation (SD). Differences were considered statistically significant when *p* < 0.05.

## 3 Results

### 3.1 Blood Glucose Levels

**Figure 1:**
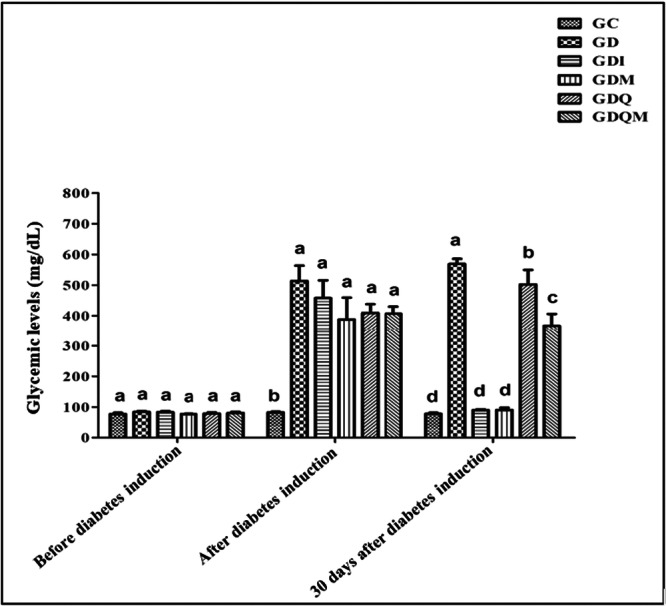
Blood glucose levels (mg/dL). GC, control group; GD, diabetic group; GDI, diabetic group treated with insulin; GDM, diabetic group treated with melatonin; GDQ, diabetic group treated with quercetin; GDQM, diabetic group treated with quercetin and melatonin. *Means followed by the same letter do not differ significantly from each other by the Tukey and Kramer multiple comparison test (*p* < 0.05).

Blood glucose levels on the day before diabetes induction were similar, so the groups did not differ, and all were within normal blood glucose levels. In the post‐induction period, only the control group had normoglycemic levels, differing from all the other groups, which demonstrated blood glucose levels above 200 mg/dL, confirming diabetes in these experimental groups. On the last day of treatment, the diabetic group continued to have the highest blood glucose levels (569.25 ± 32.57 mg/dL, *p* < 0.05). The GDQ group had a reduction in blood glucose level (502.50 ± 94.20 mg/dL, *p* < 0.05). The GDQM group showed a drop in these levels (367.00 ± 77.15 mg/dL, *p* < 0.05), falling slightly below the previous group. However, the groups with the lowest and similar blood glucose levels were the GC (87.50 ± 7.14 mg/dL, *p* < 0.05), GDI (89.50 ± 4.92 mg/dL, *p *< 0.05), and GDM (90.75 ± 3.88 mg/dL, *p* < 0.05) groups (Figure 1).

### 3.2 Liver Weights

**Figure 2:**
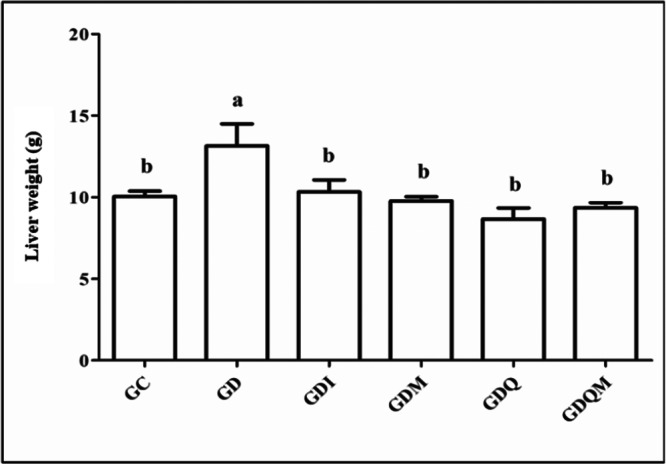
Graph of the liver weight values of the animals in the experimental groups (g). GC, control group; GD, diabetic group; GDI, diabetic group treated with insulin; GDM, diabetic group treated with melatonin; GDQ, diabetic group treated with quercetin; GDQM, diabetic group treated with quercetin and melatonin. *Means followed by the same letter do not differ significantly from each other by the Tukey and Kramer multiple comparison test (*p* < 0.05).

Analysis of liver weight showed that the GD group had the highest mean weights (12.44 ± 1.55 g, *p* < 0.05) and differed from all other groups (Figure 2).

### 3.3 Analysis of Lipid Peroxidation (TBARS) and GSH

**Figure 3:**
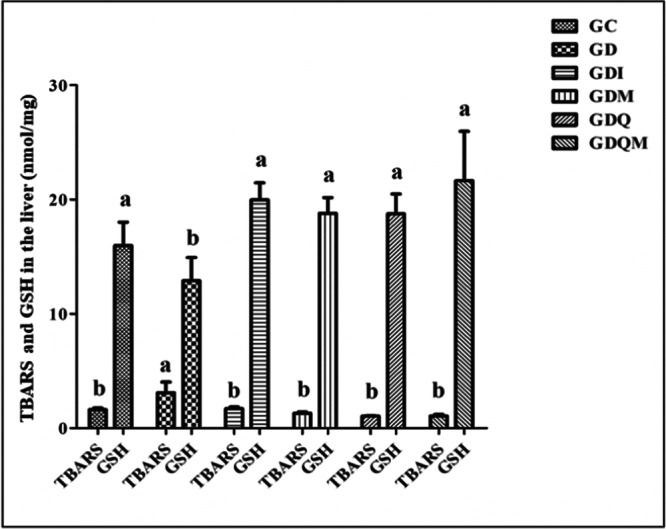
Graph of renal thiobarbituric acid‐reactive substances (TBARS) and reduced glutathione (GSH) levels (nmol/mg). GC, control group; GD, diabetic group; GDI, diabetic group treated with insulin; GDM, diabetic group treated with melatonin; GDQ, diabetic group treated with quercetin; GDQM, diabetic group treated with quercetin and melatonin. *Means followed by the same letter do not differ significantly from each other by the Tukey and Kramer multiple comparisons test (*p* < 0.05).

Analysis of oxidative stress showed that the thiobarbituric acid reactive substances (TBARS) index in the GD group presented the highest levels (3.10 ± 0.93 nmol/mg, *p* < 0.05), differing from all other groups. The other groups presented lower levels, which did not differ from each other. GSH levels presented the lowest values in the GD group (12.90 ± 1.03 nmol/mg, *p* < 0.05), differing statistically from all other groups. The GC (16.97 ± 2.04 nmol/mg, *p* < 0.05), GDI (19.97 ± 2.47 nmol/mg, *p* < 0.05), GDM (18.80 ± 1.36 nmol/mg, *p* < 0.05), GDQ (18.75 ± 1.73 nmol/mg, *p* < 0.05), and GDQM (20.65 ± 4.31 nmol/mg, *p* < 0.05) groups showed higher levels of this antioxidant and were statistically similar (Figure 3).

### 3.4 Liver Histopathology

**Figure 4:**
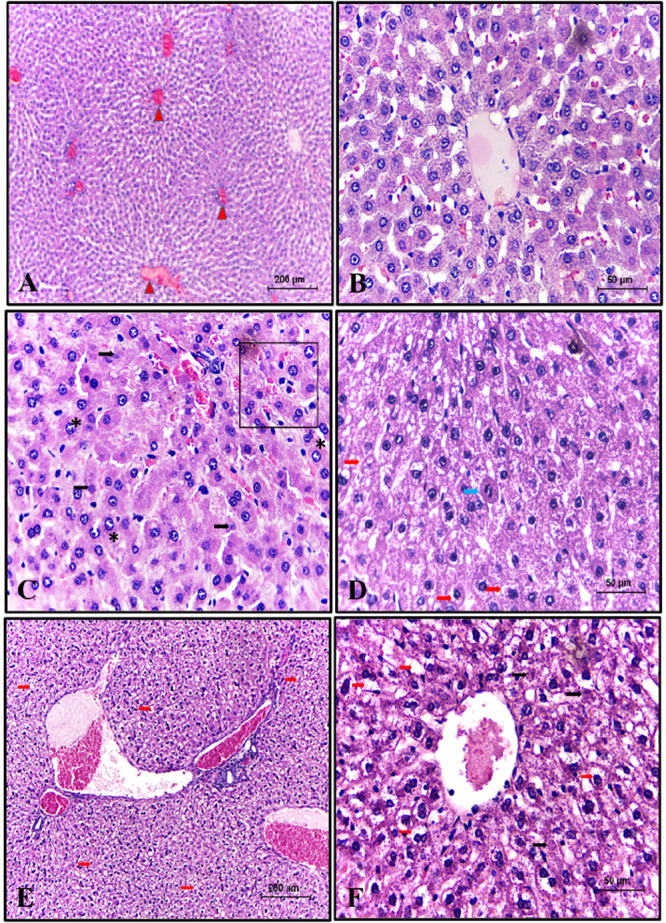
Photomicrographs of the Liver histopathology. Note in (A, B): group GC; (C, D): group GD; (E, F): group GDI. Legend of alterations: vascular congestion (red arrowhead), necrosis (black arrow), hydropic degeneration (red arrow), hepatocyte hypertrophy (asterisk), mitosis (blue arrow), and anisokaryosis (square frame). H.E. Scale bar = 50 μm.

The CG demonstrated preserved cellular and tissue structural components of the liver. The only visible alteration was mild, punctual congestion of some centrilobular veins. In the GD group (Figure 4C,D), diffuse and marked hepatocyte hypertrophy and moderate anisokaryosis were observed in most animals. Furthermore, individual and random hepatocyte necrosis was observed. Other less frequent findings included moderate to severe massive hydropic degeneration and frequent mitoses. However, in the GDI group (Figure 4E,F), massive and marked hydropic degeneration of hepatocytes was observed, along with multifocal areas of marked coagulative necrosis. Less frequent findings included diffuse and marked hepatocyte hypertrophy, as well as mild microvacuolar degeneration and multifocal areas of marked coagulative necrosis of these hepatocytes.

**Figure 5:**
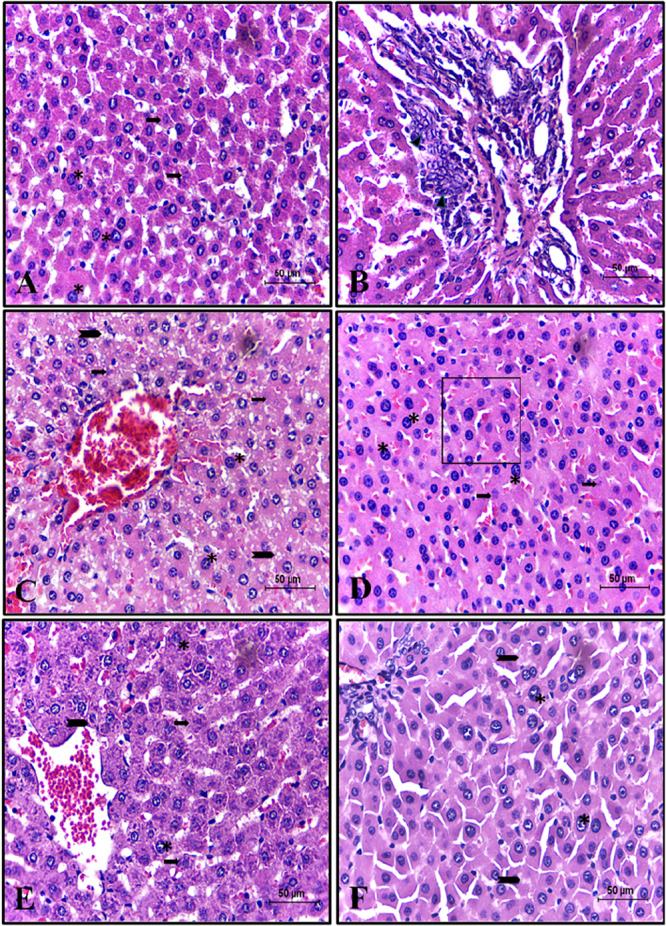
Photomicrographs of the Liver histopathology. Note in (A, B): GDM group; (C, D): GDQ group; (E, F): GDQM group. Legend of alterations: vacuolar degeneration (thick arrow), necrosis (black arrow), bile duct proliferation (black arrowhead), hepatocyte hypertrophy (asterisk), and anisokaryosis (square frame). H.E. Scale bar = 50 μm.

In the GDM group (Figure 5A,B), the most frequent findings were hepatocyte hypertrophy and areas of multifocal coagulation necrosis, all of a mild nature. A less frequent finding in this group was bile duct proliferation, which was also mild. The most frequent histopathological findings in the GDQ group (Figure 5C,D) were hepatocyte hypertrophy, as well as areas of moderate multifocal coagulation necrosis. Other less frequent alterations included mild hepatocyte vacuolar degeneration, mild bile duct proliferation, and moderate anisokaryosis. The GDQM group (Figure 5E,F) presented mild hepatocyte hypertrophy, as well as focal and mild microvacuolar degeneration and areas of moderate multifocal coagulation necrosis.

### 3.5 Liver Morphometry

Liver morphometry revealed that the GD group showed an increase in the percentage of lobular parenchyma (95.00 ± 0.90, *p* < 0.05) and a reduction in non‐lobular parenchyma (5.00 ± 0.45, *p* < 0.05). The other groups did not differ from each other with respect to these parameters (Table 1).

**Table 1:** Mean ± standard deviation of the percentage of lobular and non‐lobular parenchyma in the liver of animals in the experimental groups.

Groups	Lobular parenchyma	Non‐lobular parenchyma
GC	88.08 ± 1.11b	11.92 ± 1.11a
GD	95.00 ± 0.90a	5.00 ± 0.45b
GDI	87.67 ± 2.32b	12.33 ± 2.32a
GDM	87.83 ± 2.33b	12.17 ± 2.31a
GDQ	90.42 ± 2.30b	9.58 ± 2.04a
GDQM	85.75 ± 2.14b	14.25 ± 3.14a
*p*	0.0101	0.0103

*Note:* Means followed by the same letter in the columns do not differ significantly from each other according to the Tukey and Kramer test (*p* < 0.05).

### 3.6 Biochemical Analysis

The results of biochemical analyses of liver enzymes after 10 days of treatment revealed that the groups with the highest AST levels were DG (143.90 ± 32.22, *p* < 0.05) and GDI (129.81 ± 27.19, *p* < 0.05), which presented similar levels but different from the others. The GC (61.83 ± 2.85, *p* < 0.05), GDM (69.62 ± 6.55, *p* < 0.05), and GDQM (64.57 ± 2.67, *p* < 0.05) groups presented the lowest levels of this enzyme. The GDQ group (85.46 ± 3.70, *p* < 0.05) presented intermediate levels of this enzyme. For AST, the group with the highest levels was the GD group (91.60 ± 4.74, *p* < 0.05), differing from all other groups. The lowest levels were found in the GC (13.06 ± 5.39, *p* < 0.05), GDM (16.30 ± 4.50, *p* < 0.05), and GDQM (25.63 ± 7.15, *p* < 0.05) groups. The GDI group presented slightly lower values than the DG group, but higher than the other groups (Table 2).

**Table 2:** Mean ± standard deviation of serum levels of AST and ALT in animals in the experimental groups, after 10 days of treatment.

Groups (*n* = 4)	AST (U/L)	ALT (U/L)
GC	61.83 ± 2.85c	13.06 ± 5.39 d
GD	143.90 ± 32.22a	91.60 ± 4.74a
GDI	129.81 ± 27.19a	54.25 ± 12.47b
GDM	69.62 ± 6.55c	16.30 ± 4.50 d
GDQ	85.46 ± 3.70b	39.72 ± 3.35c
GDQM	64.57 ± 2.67c	25.63 ± 7.15 d
*p*	0.0050	< 0.0001

*Note:* Means followed by the same letter in the columns do not differ significantly from each other according to the Tukey and Kramer test (*p* < 0.05).

In the same analysis, after 30 days of treatment, we observed that the group with the highest AST levels was the DG group (88.95 ± 11.45, *p* < 0.05), which differed from all other groups. The other groups GC (51.43 ± 8.28, *p* < 0.05), GDI (48.22 ± 18.43, *p* < 0.05), GDM (54.35 ± 7.60, *p* < 0.05), GDQ (57.68 ± 7.68, *p* < 0.05), and GDQM (61.56 ± 5.19, *p *< 0.05) showed low levels of this enzyme and did not differ from each other. The same behavior between the groups occurred for ALT (Table 3).

**Table 3:** Mean ± standard deviation of serum levels of AST and ALT in animals in the experimental groups, after 30 days of treatment.

Groups (*n* = 4)	AST (U/L)	ALT (U/L)
GC	51.43 ± 8.28b	19.70 ± 6.37b
GD	88.95 ± 11.45a	68.38 ± 1.79a
GDI	48.22 ± 18.43b	18.41 ± 3.52b
GDM	54.35 ± 7.60b	19.36 ± 3.82b
GDQ	57.68 ± 7.68b	21.37 ± 5.15b
GDQM	61.56 ± 5.19b	22.22 ± 4.46b
*p*	0.0075	< 0.0001

*Note:* Means followed by the same letter in the columns do not differ significantly from each other according to the Tukey and Kramer test (*p* < 0.05).

### 3.7 Immunohistochemical Analysis (IL‐6, IL‐10, and TNF‐α)

**Figure 6:**
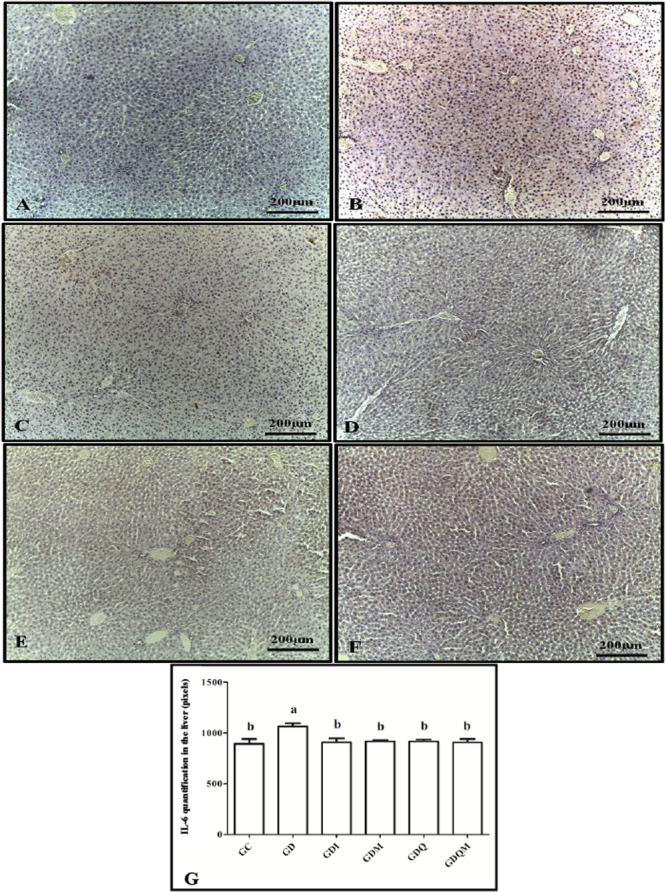
Photomicrographs of immunohistochemistry for IL‐6 in the liver of animals in the experimental groups. (A) GC, (B) GD, (C) GDI, (D) GDM, (E) GDQ, (F) GDQM, and (G) graph of the quantification in pixels of the positive brown staining of IL‐6. Means followed by the same letter do not differ significantly from each other according to the Tukey and Kramer Multiple Comparisons test (*p* < 0.05).

In the immunohistochemistry for IL‐6, it was observed that the group that presented the highest level of expression for this interleukin was the GD group, which differed from all the other groups. The GC, GDI, GDM, GDQ, and GDQM groups presented lower expression rates of this protein and were statistically similar to each other (Figure 6).

**Figure 7:**
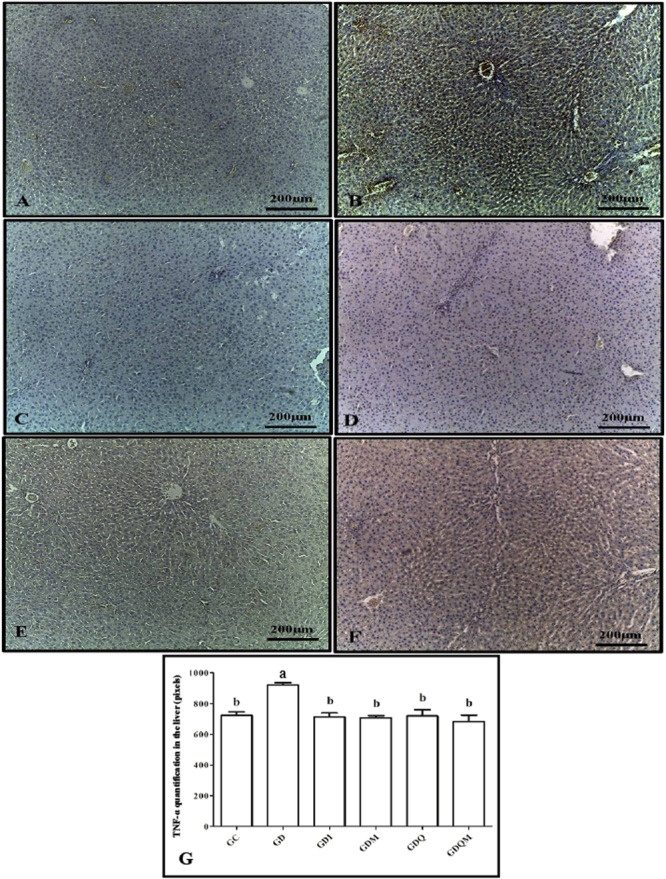
Photomicrographs of immunohistochemistry for TNF‐α in the liver of animals in the experimental groups. (A) GC, (B) GD, (C) GDI, (D) GDM, (E) GDQ, (F) GDQM, and (G) graph of the quantification in pixels of the positive brown staining of TNF‐α. Means followed by the same letter do not differ significantly from each other according to Tukey and Kramer's Multiple Comparisons test (*p* < 0.05).

The results of TNF‐α expression through the immunohistochemistry technique demonstrated that the GD group had the highest rate of expression of this interleukin, which differed from the remaining groups. However, all other groups (GC, GDI, GDM, GDQ, and GDQM) presented lower rates and did not differ statistically from each other (Figure 7).

**Figure 8:**
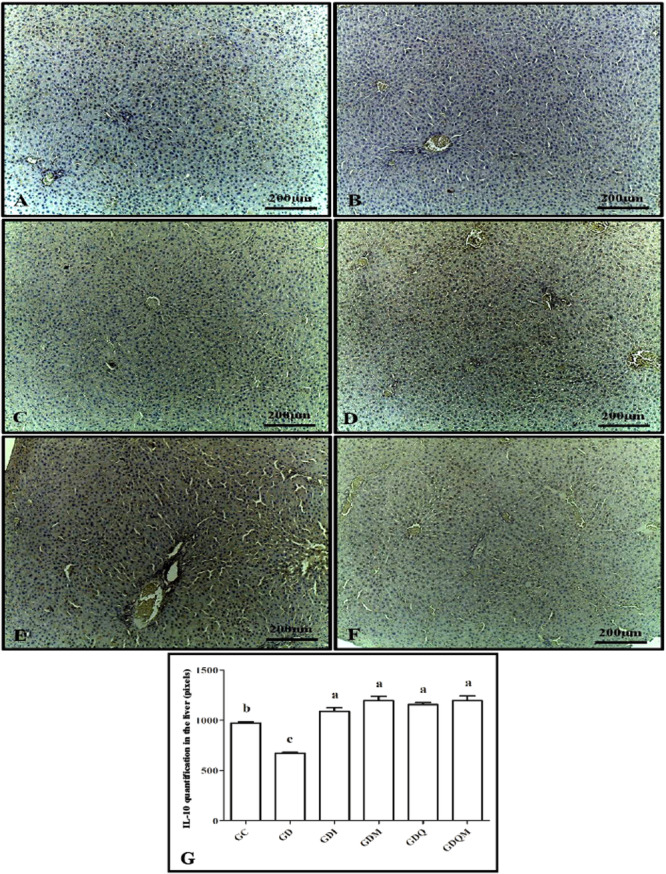
Photomicrographs of immunohistochemistry for IL‐10 in the liver of animals in the experimental groups. (A) GC, (B) GD, (C) GDI, (D) GDM, (E) GDQ, (F) GDQM, and (G) graph of the quantification in pixels of the positive staining in brown of IL‐10. Means followed by the same letter do not differ significantly from each other according to Tukey and Kramer's Multiple Comparisons test (*p* < 0.05).

Immunohistochemistry for IL‐10 revealed that the groups with the highest expression percentages were the GDI, GDM, GDQ, and GDQM groups, and they did not differ from each other. The GD group expressed the lowest amount of interleukin and was statistically isolated from the other groups. The GC group presented an intermediate level of this protein (higher than the GD and lower than the others) and differed statistically from the other experimental groups (Figure 8).

## 4 Discussion

Hyperglycemia is recognized as the main metabolic alteration responsible for diabetic complications and represents the primary cause of liver injury in diabetic individuals. In this context, the liver becomes highly susceptible to oxidative stress and inflammatory processes induced by elevated blood glucose levels [45]. At the molecular level, persistent hyperglycemia activates metabolic pathways such as the polyol pathway, advanced glycation end‐product (AGE) formation, and protein kinase C activation, culminating in excessive reactive oxygen species (ROS) production and impairment of insulin signaling through the IRS‐PI3K‐Akt axis [46].

In the present study, the groups treated with melatonin, alone or in combination, showed reduced glycemic levels. This effect may be explained by melatonin's ability to regulate energy expenditure and glucose and lipid metabolism, in addition to improving glucose tolerance [47]. Animals treated with quercetin also demonstrated a moderate reduction in glycemia, consistent with reports that this flavonoid improves glucose intolerance and modulates carbohydrate metabolism [48], possibly by preserving pancreatic β‐cell function and enhancing GLUT‐4‐mediated peripheral glucose uptake [49].

The metabolic imbalance produced by persistent hyperglycemia is closely associated with inflammatory activation and oxidative stress, both major triggers of diabetic complications [7, 50]. Excess ROS activates pro‐inflammatory transcription factors such as NF‐κB, promoting cytokine release and establishing a self‐sustaining cycle between inflammation and oxidative damage [51]. Accordingly, treatments with melatonin and quercetin, isolated or combined, reduced inflammatory cytokines IL‐6 and TNF‐α. The reduction of these mediators is essential since their elevation directly contributes to the development of diabetic complications [7]. One explanation for the anti‐inflammatory effect of melatonin is its ability to inhibit the NF‐κB signaling cascade, thereby decreasing cytokine expression [52]. Quercetin also exerts anti‐inflammatory effects by negatively regulating inflammatory pathways and reducing IL‐6, TNF‐α, and IL‐1β secretion in macrophages and fibroblasts [19]. Furthermore, quercetin may increase anti‐inflammatory cytokines, corroborating our findings of elevated IL‐10 levels in the quercetin‐treated group, an important marker of inflammatory improvement [19].

Because inflammation and oxidative stress are interconnected processes in diabetes, the antioxidant status was also affected. Quercetin, a natural antioxidant, reduces oxidative stress by activating free radical scavenging pathways and antioxidant enzymes [53, 54], explaining the decrease in TBARS and increase in GSH observed in quercetin‐treated groups. This effect is associated with activation of the Nrf2/HO‐1 pathway, which increases antioxidant enzyme expression and glutathione synthesis [20]. Melatonin also showed potent antioxidant activity due to its ability to directly neutralize oxygen‐ and nitrogen‐derived radicals and stimulate antioxidant enzyme synthesis [55], in addition to modulating mitochondrial function and reducing electron leakage in the respiratory chain [18]. Thus, diabetic animals treated with melatonin exhibited decreased TBARS and increased GSH levels.

The more pronounced increase in GSH levels observed in the combined treatment likely results from synergistic antioxidant mechanisms between melatonin and quercetin. Melatonin acts both as a direct free radical scavenger and as a modulator of antioxidant enzyme expression, restoring redox balance in streptozotocin‐induced diabetes [56] and stimulating glutathione‐dependent defense pathways [24]. Quercetin, in turn, activates antioxidant response proteins such as Nrf‐2/HO‐1 [57]. At the molecular level, melatonin stimulates GSH synthesis while quercetin reduces its oxidation by decreasing mitochondrial ROS generation, promoting preservation of the intracellular thiol pool [58]. Experimental studies demonstrate that both compounds significantly increase GSH levels and reduce MDA, promoting greater protection compared to isolated treatments [59]. Therefore, the combination likely increases glutathione availability, as melatonin stimulates its synthesis and regeneration while quercetin reduces its consumption by neutralizing reactive species.

The reduction of oxidative and inflammatory damage helps explain the improvement observed in liver morphology. Animals treated with antioxidants showed considerably less severe histological alterations than untreated diabetic animals. Morphometric analysis corroborated these findings, demonstrating that the diabetic group presented increased lobular parenchyma due to massive hepatocyte hypertrophy, causing these cells to occupy greater space, an alteration frequently associated with mitochondrial dysfunction and intracellular lipid accumulation [60]. In treated groups, parenchymal proportions remained closer to normal, indicating attenuation of cellular edema and structural damage.

The apparent absence of a relevant synergistic effect of the combination on inflammatory markers can also be understood by the partial convergence of the molecular mechanisms involved. Both melatonin and quercetin reduce inflammation mainly through modulation of the redox‐inflammatory Nrf2/NF‐κB axis [61, 62]. Antioxidant compounds that activate Nrf2 reduce the transcription of pro‐inflammatory cytokines by antagonizing NF‐κB activation, establishing a physiological limit for inflammatory suppression [51]. Thus, when administered together, the two compounds act on the same regulatory pathway, producing a more evident antioxidant effect, but not necessarily an additional reduction in inflammatory cytokines, characterizing a biological ceiling effect of the inflammatory pathway. Therefore, the combination enhances the cellular antioxidant state without proportionally amplifying the anti‐inflammatory response, which explains the absence of statistical difference between combined and isolated treatments in inflammatory parameters.

These structural improvements are consistent with normalization of liver biochemical markers such as AST and ALT, which approached control values at the end of treatment. Considering that diabetes‐induced organ damage is sustained by chronic inflammatory and oxidative cascades [7, 50], attenuation of these processes directly contributes to hepatic protection and functional recovery, possibly through preservation of mitochondrial integrity and intracellular redox signaling [63, 64].

It is crucial to acknowledge the limitations of the present study. The short treatment period (30 days) does not allow for the assessment of chronic effects or long‐term safety of the combined therapy. The absence of a dose‐response evaluation for both compounds limits the understanding of their pharmacodynamics. The analysis of oxidative stress was restricted to GSH and TBARS markers, and liver function was assessed only by AST and ALT enzymes. Future studies should include a broader panel of cytokines and oxidative stress markers (such as catalase, superoxide dismutase) and investigate the molecular pathways involved (e.g., Nrf2, NF‐κB) more deeply, using techniques such as western blot analysis or real‐time PCR.

## 5 Conclusion

The results demonstrate that antioxidant treatment with melatonin and quercetin attenuated diabetes‐induced hepatic alterations by reducing hyperglycemia, oxidative stress, and inflammatory responses, while preserving hepatic architecture. The combined therapy showed a particularly relevant effect on the endogenous antioxidant system, especially by improving GSH levels. Although promising, these findings require further studies to elucidate the underlying molecular mechanisms and to evaluate the potential of these compounds as adjuvant therapies in the management of diabetes mellitus.

## Author Contributions

**Érique Ricardo Alves:** conceptualization, investigation, writing – original draft, methodology, validation, visualization, writing – review and editing. **Jaiurte Gomes Martins da Silva:** writing – original draft, writing – review and editing, methodology. **Ismaela Maria Ferreira de Melo:** investigation, writing – original draft, methodology, writing – review and editing. **Laís Caroline da Silva Santos:** methodology, investigation, writing – original draft, writing – review and editing. **Marcelle Mariana Sales de França:** writing – original draft, writing – review and editing, methodology. **Clovis Lapa Neto:** writing – original draft, methodology, writing – review and editing. **Vanessa Bischoff Medina:** writing – review and editing, writing – original draft, methodology. **Francisco de Assis Leite Souza:** methodology, writing – review and editing, writing – original draft. **Leucio Duarte Vieira:** writing – original draft, writing – review and editing, methodology. **Álvaro Aguiar Coelho Teixeira:** conceptualization, investigation, funding acquisition, writing – original draft, methodology, validation, visualization, writing – review and editing, formal analysis, project administration, supervision, resources, data curation. **Valéria Wanderley Teixeira:** conceptualization, investigation, funding acquisition, writing – original draft, methodology, validation, visualization, writing – review and editing, formal analysis, project administration, resources, supervision, data curation.

## Data Availability

The data that support the findings of this study are available on request from the corresponding author. The data are not publicly available due to privacy or ethical restrictions.
